# The Time Course of Injury Risk After Return-to-Play in Professional Football (Soccer)

**DOI:** 10.1007/s40279-024-02103-3

**Published:** 2024-09-14

**Authors:** Guangze Zhang, Michel Brink, Karen aus der Fünten, Tobias Tröß, Peter Willeit, Tim Meyer, Koen Lemmink, Anne Hecksteden

**Affiliations:** 1 Institute of Sports and Preventive Medicine, Saarland University, Saarbrücken, Germany; 2 Center for Human Movement Sciences, University of Groningen, University Medical Center Groningen (UMCG), Groningen, The Netherlands; 3 University Sports, Stuttgart University, Stuttgart, Germany; 4 Institute of Health Economics, Medical University of Innsbruck, Innsbruck, Austria; 5 Department of Public Health and Primary Care, University of Cambridge, Cambridge, UK; 6 Department of Sport Science, Institute of Sport Science, University of Innsbruck, Fürstenweg 176, 6020 Innsbruck, Austria; 7 Institute of Physiology, Medical University of Innsbruck, Innsbruck, Austria

## Abstract

**Background:**

Injury risk in professional football (soccer) is increased in the weeks following return-to-play (RTP). However, the time course of injury risk after RTP (the hazard curve) as well as its influencing factors are largely unknown. This knowledge gap, which is arguably due to the volatility of instantaneous risk when calculated for short time intervals, impedes on informed RTP decision making and post-RTP player management.

**Objectives:**

This study aimed to characterize the hazard curve for non-contact time-loss injuries after RTP in male professional football and to investigate the influence of the severity of the index injury and playing position.

**Methods:**

Media-based injury records from the first German football league were collected over four seasons as previously published. Time-to-event analysis was employed for non-contact time-loss injury after RTP. The Kaplan–Meier survival function was used to calculate the cumulative hazard function, from which the continuous hazard function was retrieved by derivation.

**Results:**

There were 1623 observed and 1520 censored events from 646 players analyzed. The overall shape of the hazard curve was compatible with an exponential decline of injury risk, from an approximately two-fold level shortly after RTP towards baseline, with a half-time of about 4 weeks. Interestingly, the peak of the hazard curve was slightly delayed for moderate and more clearly for severe index injuries.

**Conclusions:**

The time course of injury risk after RTP (the hazard curve) can be characterized based on the Kaplan–Meier model. The shape of the hazard curve and its influencing factors are of practical as well as methodological relevance and warrant further investigation.

**Supplementary Information:**

The online version contains supplementary material available at 10.1007/s40279-024-02103-3.

## Key Points

**Table:** 

Continuous hazard curves characterize the risk trajectory of subsequent non-contact injuries, showing a decline towards baseline within 4 weeks after return-to-play, with delayed risk peaks for severe injuries and forwards.
The “one-month excess risk decay” facilitates return-to-play decision making and post-return-to-play player management, and informs the follow-up period in epidemiological studies.

## Introduction

Injuries are common in professional football players and seemingly unavoidable in their career [1]. As players return-to-play (RTP) from a previous injury, the risk of subsequent injury is high [2] and the timepoint at which the risk returns to baseline is largely unknown. Given the economic and competitive implications of injury burden, decisions on the timing of RTP and post-RTP player management are of particular importance for stakeholders (e.g., coaches, players, and managers) [3]. Although there have been attempts to determine the time period within which injury risk is elevated [4–7], the time course of excess injury risk after RTP (in more technical terms: the trajectory of instantaneous injury risk over time) has yet to be determined.

Regarding the interval between RTP and subsequent injury, 50–80% of subsequent injuries are reported to occur within the first 4 weeks [4, 7]. However, those figures are based on injury frequency and do not consider the fact that the number of players still at risk (i.e., the cohort that have not sustained a subsequent injury) also drops as time goes by because each injured player reduces this group. A decreasing number of players at risk would lead to a decline of injury occurrence even with constant injury risk. Time-to-event analysis [8] (survival analysis) has been widely employed to investigate the occurrence and timing of events in epidemiology [9] and beyond [10]. In the context of injury occurrence, time-to-event analysis (using the Kaplan–Meier method [11]) allows for considering the fact of decreasing number of players at risk. Previous studies have generally quantified injury risk after RTP in coarse time intervals of 2–8 weeks [4, 7, 12]. In such a discrete time interval (e.g., weeks) [13], injury risk for a specific time interval can be conveniently estimated by dividing the number of injuries by the number of players still at risk [13]. However, discrete hazard estimation with a coarse time metric (e.g., months) may be inadequate to capture the rapid changes in injury risk after RTP. The key challenge for a fine-grained time scale (e.g., days) is the volatility of hazard estimates due to the low number of injuries per time interval (including intervals without injury at all). As a consequence, the hazard function cannot be directly estimated over a continuous timeline [13]. The present work explores the application of an established statistical solution [13] within the field of injury risk after RTP.

In the analysis of injury occurrences and RTPs over a football career, two classes of time intervals must be distinguished. The interval between an (index) injury and RTP (i.e., RTP time) and the interval between RTP and a subsequent injury. While the present work will be focusing on the latter, RTP time is also of interest for the following two reasons. First, according to the time-loss concept, time to RTP is used as an indicator of injury severity [14] with implications for post-RTP injury risk [15]. The respective consensus has been widely applied [7, 16]. Second, RTP time relative to the initial diagnosis of the injury could allow an estimation on rehabilitation (in)adequacy, which also influences injury risk after RTP [6, 17]. In addition, the playing position is also an established influencing factor of injury risk within and beyond the context of RTP [1].

In existing time-to-injury analysis in football, Cox regression models have been widely used to estimate discrete hazard ratios for supposed risk factors [17], including in the RTP context [7]. However, a fundamental assumption of the Cox model (proportional hazard) has not been verified so far. That is, that hazard functions for these risk factors are proportional (parallel hazard curves over time [18]) and their association can therefore be summarized as one common hazard ratio.

Contact injuries are relatively unpredictable in football [19]. Therefore, the present work aims to characterize the continuous-time hazard curve of non-contact injuries after RTP in professional football. Moreover, variations of the continuous hazard curve among severities of index injury and playing positions will be investigated. The hazard curves may also verify whether the proportional assumptions are met.

## Methods

### Dataset

The analyzed data included four seasons (2014/15 to 2017/18) of media-based injury records in the first male German football league, a subset of the dataset used by Aus der Fünten et al. [20]. Injuries were prospectively documented in a standardized manner through an official collaboration with German kicker^®^ sports magazine [21–24], with team-specific updates provided by assigned journalists. Additionally, injuries were daily monitored through the social media websites of teams and players, as well as the online platform https://ligainsider.de, with occasional reference to http://www.transfermarkt.de. Each injury entry in the database was verified by at least one additional source, and diagnoses were confirmed by medical staff according to international guidelines [14]. Neither research ethics board approval nor a trial registration were required as all data were collected from publicly available sources.

### Equity, Diversity, and Inclusion Statement

The focus of this work is on male professional football. While the specific results are presumably dependent on discipline, performance level, and sex, the method presented may be applied in other settings and populations.

### Data Analysis

#### Pre-processing

A RTP scenario is triggered by an index injury followed by rehabilitation, RTP, and eventually a subsequent injury. As contact and non-contact injuries can equally impact on players’ physical condition and subsequently influence the injury risk after RTP, both categories are considered for the index injury. However, given the unpredictability of physical contact in football, only non-contact time-loss injuries were considered as subsequent injuries. For each player, the first injury on record was considered as the index injury of the following injury, the second injury as the index injury of the third injury. Return-to-play in this study was defined as a full return to training and competition [7].

The severity of index injuries was categorized according to the time loss concept: minimal (1–3 days), mild (4–7 days), moderate (8–28 days), and severe (> 28 days) [14]. The playing position was considered as the players’ main position when the subsequent injury occurred, including goalkeeper, defender, midfielder, and forward. All data processing and following analysis was performed using R Statistical Software (v4.2.2; R Core Team 2022). The knit R Markdown files for data analysis have been made available in the following repository (https://github.com/latilongitude/Injury_risk_after_RTP).

#### Censoring

**Fig. 1:**
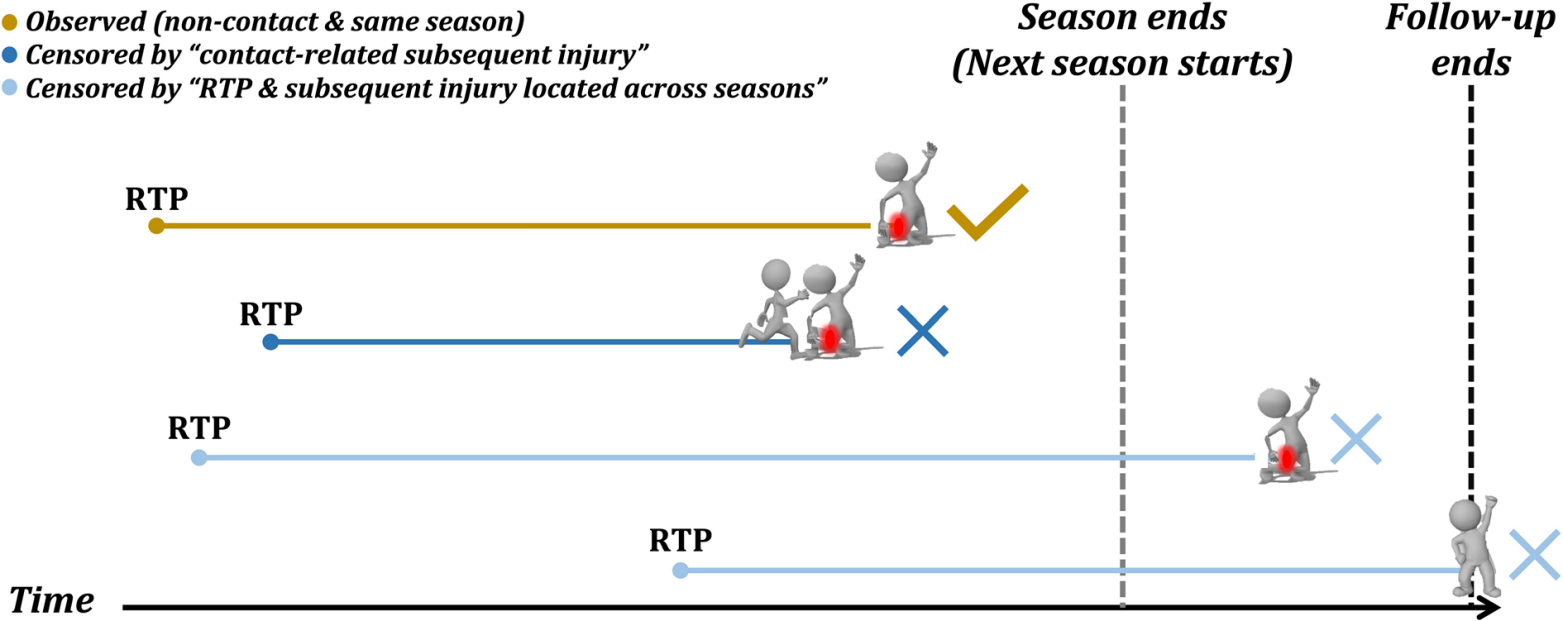
Two strategies of censoring observations. The example at the bottom is not subsequently injured until the end of the follow-up. *RTP* return-to-play

Censoring refers to an abbreviated length of follow-up due to the end of the follow-up period or reasons other than the target event. Four football seasons were segmented by the date of the last official match for corresponding season, 23 May for 2014/15 season, 14 May for 2015/16 season, 20 May for 2016/17 season, and 12 May for 2017/18 season. Given that training and match exposure as well as the recording of minor injuries during the summer break might differ from in-season, cases that did not incur a subsequent injury in the same season as RTP were censored at the end of season (date of last match, cp. above and Fig. 1). As this study mainly focused on the occurrence of non-contact injury after RTP, contact-related subsequent injuries equally led to censoring (Fig. 1). A subsequent injury was confirmed as an observed event only when it was observed in both categories (i.e., non-contact subsequent injury occurring in the same season as RTP).

#### Hazard Function

**Fig. 2:**
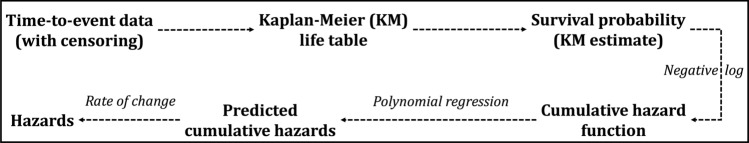
Retrieving the hazard function on continuous time. *KM* Kaplan–Meier

Figure 2 illustrates the steps to derive the continuous hazard function. First, the dataset with censoring information was used to fit a Kaplan–Meier (KM) model [8]. Because of the fine-grained time metric (day), the number of observed events might be trivial for individual time units, which makes the discrete-time KM hazards too volatile to be meaningful. By contrast, at each time ${t}_{j}$, the cumulative hazard function $\widehat{H}({t}_{j})$ can be derived through an established mathematical relationship (see Eq. 1) from the KM survival function ${\widehat{S}}_{\text{KM}}({t}_{j})$ [13]. The instantaneous risk is the change in cumulative hazard from one time unit (day) to the next, that is, the local slope of the cumulative hazard function. The cumulative hazard function provides a pivot to retrieve continuous hazards as its first derivative [13]. To simplify the calculation, cumulative hazards and time, as response and explanatory variables, respectively, were used to fit a polynomial (tenth degree) regression model. Subsequently, predictions were made for successive days. The rate of change in predicted cumulative hazards was then calculated as the continuous hazard function (i.e., the risk of subsequent injury) [13]. It is important to note that only cumulative hazards from the first 100 days after RTP were used to fit the polynomial.

1 $$\hat{H}\left( {t_{j} } \right) = - {\text{ln}}\hat{S}_{{{\text{KM}}}} \left( {t_{j} } \right).$$

A linear interpolation approach [8] was applied to estimate the median survival time $T$, as shown in Eq. 2 where $m$ represents the time interval when the sample survival function is just above 0.5.

2 $$T = m + \left[ {\frac{{\hat{S}_{{{\text{KM}}}} \left( {t_{m} } \right) - 0.5}}{{\hat{S}_{{{\text{KM}}}} \left( {t_{m} } \right) - \hat{S}_{{{\text{KM}}}} \left( {t_{m + 1} } \right)}}} \right]\left( {\left( {m + 1} \right) - m} \right).$$

#### Ancillary Analysis 1: Time Course of Injury Risk Outside of the RTP Context

As detailed in Sect. 1, injury risk is assumed to decline from elevated risks after RTP towards a stable “baseline risk” without systematic dependence on time. While this assumption is highly plausible, it could not be verified directly so far. For comparison, we therefore also applied the above-described method to derive a hazard curve for players not returning from a recent injury. This comparator is based on a time period subsequent to the first 100 days after RTP, which are used to derive the main hazard curve (see Fig. S1-1 in the ESM).

#### Ancillary Analysis 2: Considering Hierarchical Data Structure

It is important to note that the analyzed dataset features a hierarchical data structure. As players with more frequent injuries (and therefore RTPs) contribute more data points (see Figs. S2-1 and S2-2 of the ESM), these individuals have a disproportionately high impact on the hazard function, leading to potential bias. Aiming to expose the general analytical pipeline for deriving the continuous hazard function as transparently as possible, the nesting of RTP episodes within players is not considered in the above analysis. However, in the ESM, we illustrate and discuss up-sampling and down-sampling, respectively, of the original dataset as two potential options for mitigating this issue while still avoiding advanced modelling techniques.

#### Ancillary Analysis 3: Round-Robin Splitting to Check Overfitting

To address concerns about overfitting by high-degree polynomial regression, we performed a leave-one-quarter-out data splitting in a round-robin manner. Splitting was performed on the player level and stratified for playing positions to avoid information leakage. The resulting hazard curves were compared with those from the main analysis to probe potential overfitting (see Fig. S3-1 in the ESM).

## Results

### Epidemiology of Subsequent Injuries

Within the four seasons, 822 players incurred 4065 injuries, with a total of 3143 subsequent injuries involving 646 players. Six hundred and seventy-four (21.4%) subsequent injuries occurred across the end of season, which in conjunction with contact-related injuries (*n* = 1102, 35.1%) resulted in 1520 censored cases and 1623 observed subsequent injuries.

**Fig. 3:**
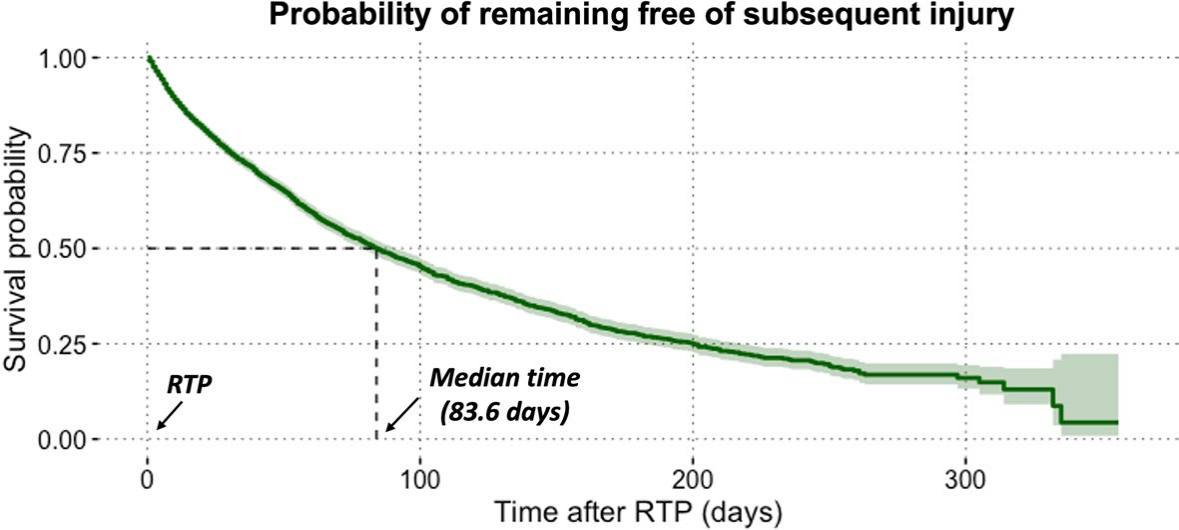
Kaplan–Meier estimates of continuous-time survivor function with the 95% confidence interval and median survival time. *RTP* return-to-play

**Fig. 4:**
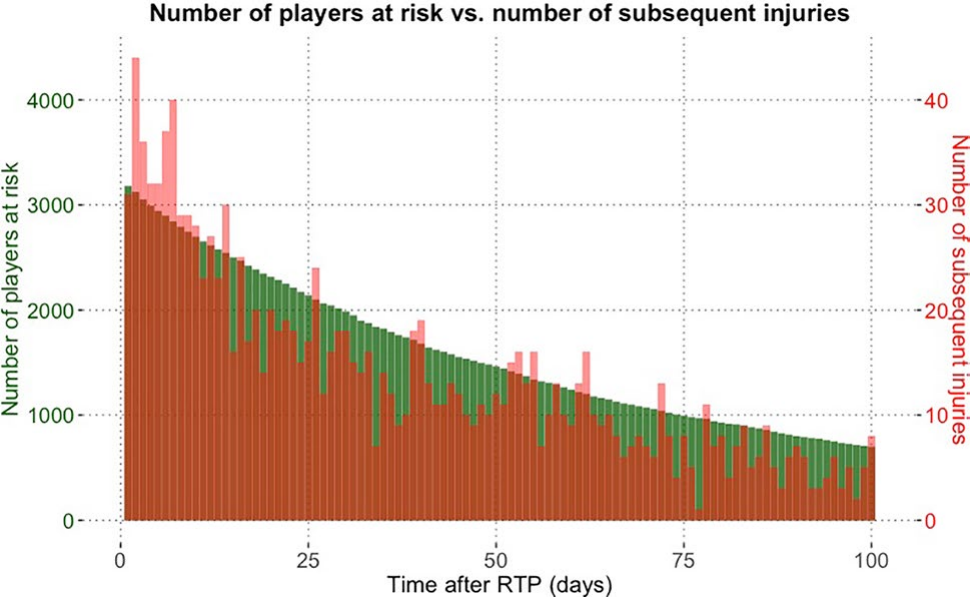
Number of players still at risk (green bar) and observed subsequent injuries (red bar) at the time course after return-to-play (RTP)

Seventy-seven percent (*n* = 2406) of all subsequent injuries and 83% (*n* = 1343) of observed subsequent injuries were sustained during the first 100 days after RTP. The median survival time was 84 days after RTP (Fig. 3). Figure 4 showed that the number of players still at risk steadily fell over the post-RTP period. Observed subsequent injuries presented a similar overall pattern, however, with fluctuation.

**Fig. 5:**
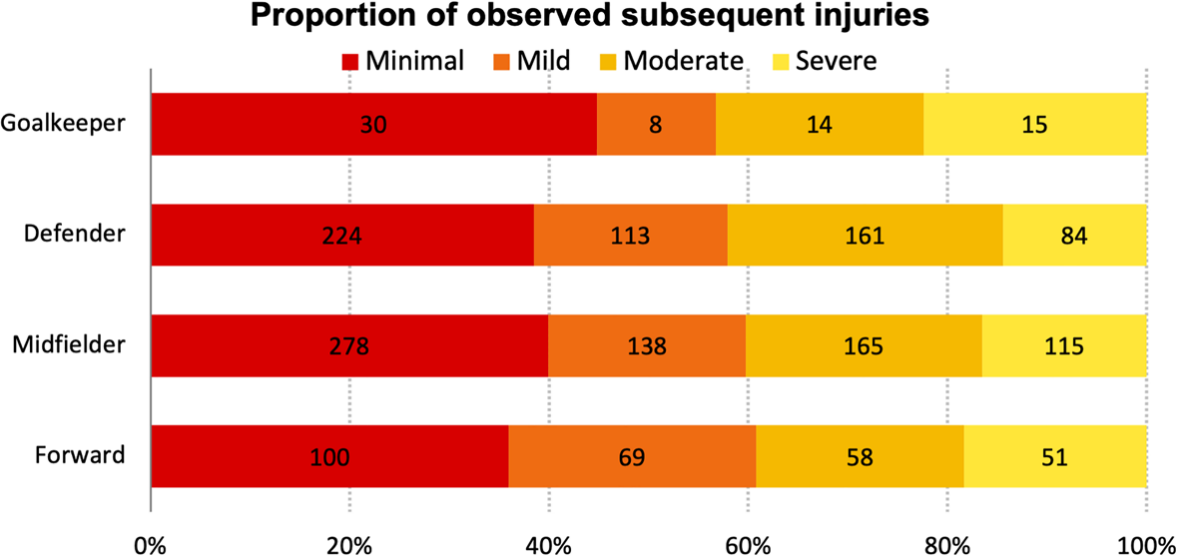
Distribution of observed subsequent injuries after different severities of index injury across playing positions

Among all observed subsequent injuries, minimal, mild, moderate, and severe index injuries contributed 38.9%, 20.2%, 24.5%, and 16.3%, respectively. The respective proportions of severity categories were similar across playing positions (Fig. 5). With respect to playing positions, 551 (including 278 observed) subsequent injuries were experienced by 141 forwards, 1406 (696 observed) subsequent injuries by 272 midfielders, 1051 (582 observed) subsequent injuries by 236 defenders, and 135 (67 observed) subsequent injuries by 41 goalkeepers.

### Risk of Subsequent Injury (Hazard Curve)

**Fig. 6:**
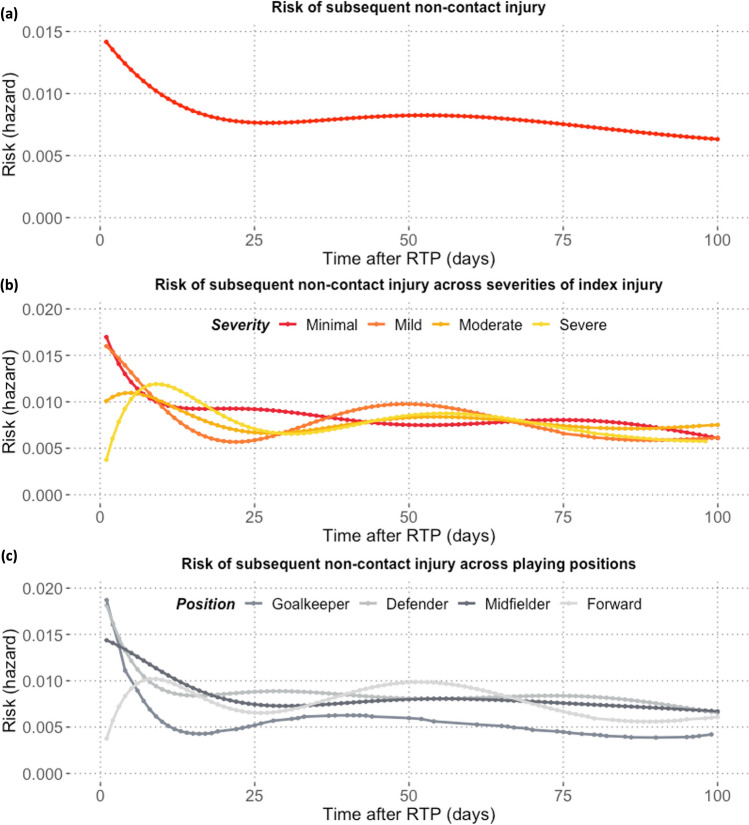
Time course of **a** non-contact injury risk after return-to-play (RTP); **b** non-contact subsequent injury risk across severities of index injury; and **c** playing positions

Overall, as players returned to play, the risk of non-contact subsequent injury was about two times higher than the baseline. Across all analyzed events, the shape of the hazard curve is compatible with an exponential decay of excess risk, which diminished by half after approximately 25 days and levels off afterwards (Fig. 6a). There is a larger relative change over time when analyzing injury frequencies (the red bars in Fig. 4) versus hazards (Fig. 6a), which take the number of players at risk into account.

As shown in Fig. 6b, the shape of the hazard curve differs across severities of the index injury. For minimal and mild index injuries, the “exponential” pattern holds. A minor risk increment over the first 5 days was observed for moderate index injuries and RTP from severe injuries was followed by a significantly increasing injury risk within the first 10 days, which thereafter remained relatively high.

Goalkeepers, midfielders, and defenders faced a decreasing risk of non-contact injuries in the first 4 weeks as they returned to full football activity (Fig. 6c). The greatest plunge in injury risk was seen in goalkeepers, with an approximate decline by 75% in the 2 weeks after RTP. In contrast, forwards experienced a slightly increasing risk of getting injured again after RTP, which fluctuated over the post-RTP period.

A comparator analysis outside the RTP context showed generally a lower level of injury risk and no systematic dependence on time (cp. ESM). It is important to note that the number of players still at risk decreased with increasing injury-free time. Therefore, the tenth degree polynomial used to fit the hazard curve after RTP likely overfitted these comparator data, plausibly explaining the noticeable variation.

Compensating for the overrepresentation of frequently injured players by either up-sampling or down-sampling the original dataset did not lead to marked changes in the overall shape of the hazard curve (cp. ESM). Moreover, round-robin data splitting showed qualitatively similar hazard curve patterns to those observed in the main analysis, supporting the validity of results (cp. ESM).

## Discussion

The primary aim of this study was to derive and investigate the hazard curves for subsequent non-contact injuries after RTP in professional football. The overall shape of the hazard curve followed the expected pattern of an exponential “decay” of excess risk towards baseline. Importantly, in contrast to previous studies [4, 7] investigating the timing of subsequent injuries after RTP, the current study determined a continuous hazard function, thereby avoiding an overestimation of changes in injury risk. With respect to the risk across severities of index injury and playing positions, as opposed to other groups in each category, a delayed maximum of hazard was found for those returning from moderate and severe index injuries and for forwards. This is indicative of a period and a group of players that need particular attention. Hazards appeared not to be proportional for these groups.

Bengtsson et al. [2] reported an increased injury rate in the first match appearance after RTP compared with the average seasonal match injury rate for all injuries (46.9 vs 25.0/1000 h) and for muscle injuries (24.6 vs 9.5/1000 h) when injury risk was simply calculated by dividing the number of injured players by the total number of players. In a 3-year follow-up study [25], six (6.7%) players after anterior cruciate ligament reconstruction experienced complications (five re-ruptures and four other knee injuries) between return-to-training and the first match. While, similarly, Orchard and Best [5] found that players in the Australian Football League faced the highest injury risk during the first week after RTP, they also reported that risk was still increased during the following weeks. This could be explained by ongoing muscle regeneration after a rehabilitation of weeks [26]. Nevertheless, these findings were obtained from incidence rates averaged over a certain period and critically depended on the follow-up duration, thereby ignoring the potentially time-varying distribution of injury over time [18, 27]. The current time-to-event analysis presented the continuous hazard of non-contact injuries after RTP and takes into account its time-varying characteristics.

While in the current study, the risks of subsequent injury steadily declined after returning from minimal and mild index injuries, rehabilitation adequacy for these cases should not be overlooked in practice [28]. Ekstrand and Gillquist [12] reported that a minimal or mild injury could be followed by a more severe subsequent injury. Severe injuries are usually less common in football but cause longer absence times [16]. The delayed peak of injury risk after returning from severe index injuries may be due to larger tissue damage, for example, damage of nerves and impaired proprioception. This might require a longer time for regeneration. Detraining effects after a long absence and immobilization due to severe injuries could also reduce players’ muscle mass [29], proprioception [16], and cardiovascular capacity [30]. Players returning from severe injuries may play with greater care in the first days. After several regular training sessions, their confidence may be restored sooner than actual athletic capacity, which in conjunction with the expectation of proving themselves may lead to the delayed peak of injury risk. Thus, players returning from severe injuries warrant further attention and monitoring over the following weeks.

The trajectory of non-contact injury risk after RTP differed across playing positions. This could correspond with the fact that different roles were exposed to various intensities of physical contact in training and match play [31, 32]. Forwards faced a delayed peak shortly after RTP with some fluctuations over the post-RTP period. Carling et al. [1] similarly found that center forwards sustained a higher incidence of recurrent muscle strains than other positions. Comparable results were reported for groin injuries [33]. Nevertheless, findings are inconsistent across studies for an association between playing positions and injury risk [34]. Some rotation of playing positions in modern football may explain the inconsistency. Classification of playing positions in this type of study may need further discussion.

In this study, the characterization of hazard curves enables a direct visual assessment of the proportional hazard assumption. Importantly, hazard curves differed in overall shape and scale across severities of index injury and playing positions. This result points to the necessity of explicitly verifying the proportional hazard assumption for any given setting. The direct visual assessment of the quantity of interest could be a pragmatic approach especially when working with limited sample sizes [35]. Previously, more indirect visualizations have been used to examine this assumption, such as survival function against time, cumulative hazard versus time, log (cumulative hazard) versus log (time), or Schoenfeld residuals versus log (-survival function) [9, 36]. For example, Della Villa et al. [37] examined the proportional hazards assumption with Schoenfeld residuals when investigating potential factors associated with second anterior cruciate ligament injuries, where the assumption was globally met for all candidate risk factors. Again, sample size and statistical power should be considered for the specific case.

### Strengths, Limitations, and Future Directions

For the first time, this study used time-to-event analysis to characterize the continuous hazard curves for subsequent non-contact injuries in football. While introducing this method to sports injury research offers qualitatively new insights, it should be noted that the retrospective media-based data set is associated with some limitations. Our analyses were limited to basic pieces of information (timepoints of injury and RTP, playing position) that are timely and reliably reported for the German Bundesliga by specialized media outlets. Considering the limitations of media-based data collection (specifically regarding injury diagnosis) as well as sample size requirements, we did not analyze hazard curves for specific index injury diagnoses or recurrences. However, this topic clearly warrants further research in follow-up studies. Moreover, exposure hours (or exposure load) after RTP could provide more accurate insights into the time course of injury risk after RTP. Future research could also consider additional influencing factors such as injury history (e.g., frequency of previous injuries) [38], rehabilitation adequacy [15], location (e.g., the body region [38, 39] and affected tissue [6, 16]), and type [1, 17] of index injury. It should be noted that while the method is applicable beyond male professional soccer, the specific results reported above are likely not transferable to other playing levels or female football, or to other sports.

Finally, as mentioned in Sect. 2.3.5, the main analysis did not consider the nesting of events within individuals. It has to be kept in mind that this might lead to bias because frequent injury occurrence leads to overrepresentation of episodes involving the concerned player in the dataset and is, at the same time, plausibly associated with shorter time intervals (injury severity and time between RTP and subsequent injuries). Respecting the proof-of-concept character of this work, we consciously opted to focus on exposing the main analytical strategy. However, in the ESM, we illustrate two potential solutions that still avoid the advanced modeling technique: (a) randomly up-sampling on the individual level within each season to the maximum number of RTPs per player in the corresponding season (see Figs. S2–3 of the ESM) and (b) including only the first RTP of each player within each season (see Figs. S2–4 of the ESM). Both methods operate on the level of data processing without requiring alterations of the main analytical proceedings presented in Sect. 2.3. Importantly, all three analytical options result in a similar overall shape of the hazard curve. Moreover, ancillary analysis 3 demonstrated qualitatively similar risk trajectories in subsets of the original data, addressing concerns about overfitting by high-degree polynomial regression. It is important to note that it would have been desirable to use independent subsets instead of a leave-one-quarter-out round-robin. However, case numbers in independent subsets were too small (see Fig. S3-1 of the ESM). Consequently, out-of-population verification of differences in risk trajectories is warranted.

## Conclusions

Through time-to-event analysis, this study determined the continuous hazard curve of non-contact injuries after RTP, which was two times higher at the day of RTP than the baseline level. One month follow-up after RTP is reasonable to capture most of the “surplus” risk of subsequent non-contact injury while avoiding excessive effort as well as “dilution” with injuries unrelated to RTP. The severity of index injury and playing position impact on the time course of the non-contact injury after RTP, resulting in a severity-dependent delay of the peak hazard. Post-RTP player management benefits from a valid estimate of the remaining excess injury risk as time elapses. Replication and further investigation are warranted before applying specific results in practice.

## Supplementary Information

Below is the link to the electronic supplementary material.

Comparing the time course of the injury risk after RTP with that of the risk outside RTP context (DOCX 460 KB)

Hierarchical data structure and two alternatives for data processing (DOCX 1242 KB)

Data splitting for addressing overfitting concern (DOCX 2631 KB)

## Declarations

### Availability of data and material

The datasets used and analyzed during the current study are available from the corresponding author on reasonable request.
