# Supplementary material for: The Time Course of Injury Risk After Return-to-Play in Professional Football (Soccer)
Source: Sports Med. 2024 Sep 14;55(1):193–201. doi: 10.1007/s40279-024-02103-3 (PMC11787231; doi:10.1007/s40279-024-02103-3)
Supplement: Supplementary file 1 — Comparing the time course of the injury risk after RTP with that of the risk outside RTP context (DOCX 460 KB) [file 40279_2024_2103_MOESM1_ESM.docx]

**Fig. S1-1** Kaplan-Meier estimates of continuous-time survivor function with 95% confidence interval and median survival time for (a) players just returning to play from an injury, and (b) players without a recent injury. The time course of non-contact injury risk for (c) players just returning to play from an injury, and (d) players without a recent injury. Two panels are provided for comparison. RTP, return to play.
