# Supplementary material for: The Time Course of Injury Risk After Return-to-Play in Professional Football (Soccer)
Source: Sports Med. 2024 Sep 14;55(1):193–201. doi: 10.1007/s40279-024-02103-3 (PMC11787231; doi:10.1007/s40279-024-02103-3)
Supplement: Supplementary file 2 — Hierarchical data structure and two alternatives for data processing (DOCX 1242 KB) [file 40279_2024_2103_MOESM2_ESM.docx]

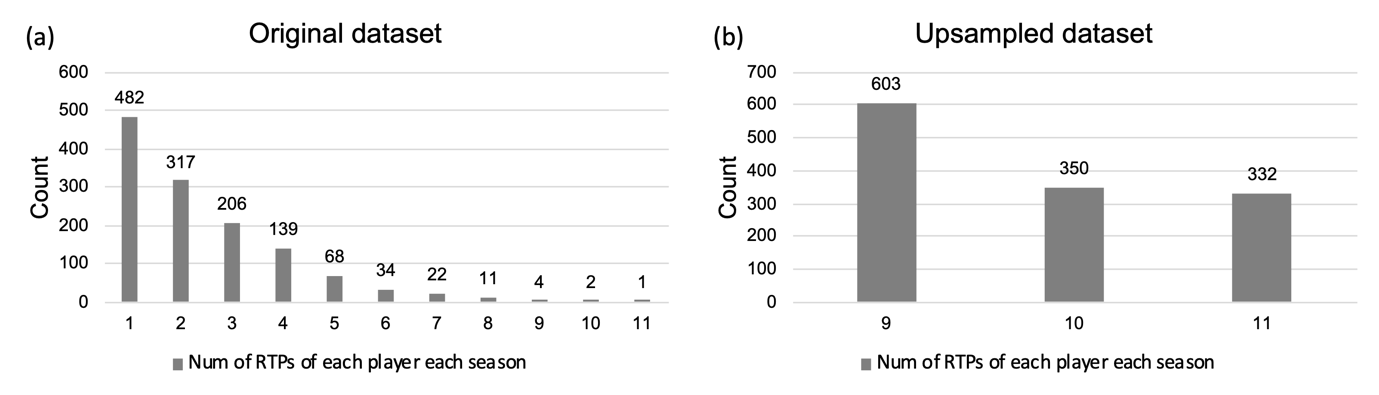


**Fig. S2-1** The number of RTPs of each player within each season in the (a) original dataset, and (b) up-sampled dataset.


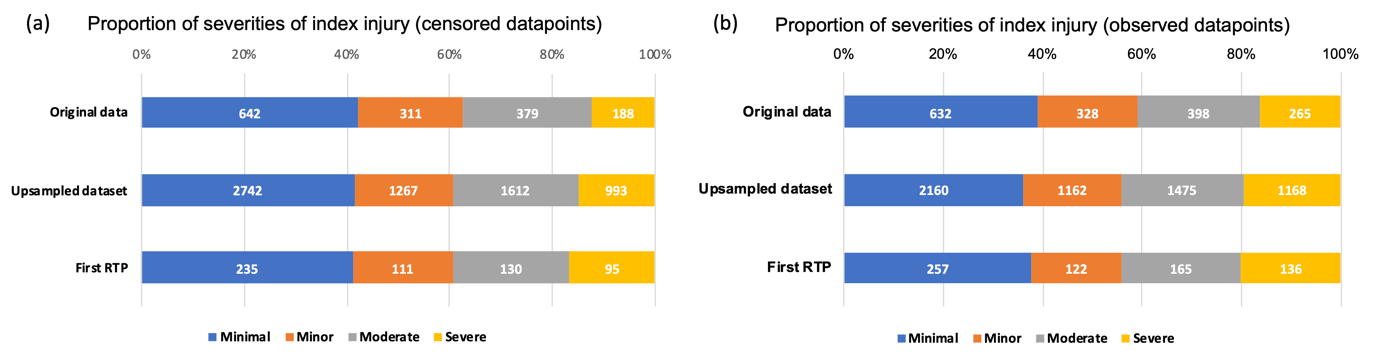


**Fig. S2-2** Distribution of severities of index injury in (a) censored, and (b) observed data points, across datasets.

**Alternative data-processing 1: Randomly up-sampling on the individual level**

Here, the number of RTP episodes per player and season was balanced by random up-sampling on the individual level. Specifically, the RTP episodes originally recorded for a specific player within a season were amended by randomly drawing from this set until reaching the maximum number of RTPs per player in the corresponding season. For example, a maximum of 9 subsequent injuries from individuals was found for season 2014/15. For this season, injury records of players who sustained less than 9 subsequent injuries were amended by randomly drawing from the originally observed RTP episodes of the respective player within the 2014/15 season. The number of up-sampled injury records at individual level can be found in Fig. S-1b.

**
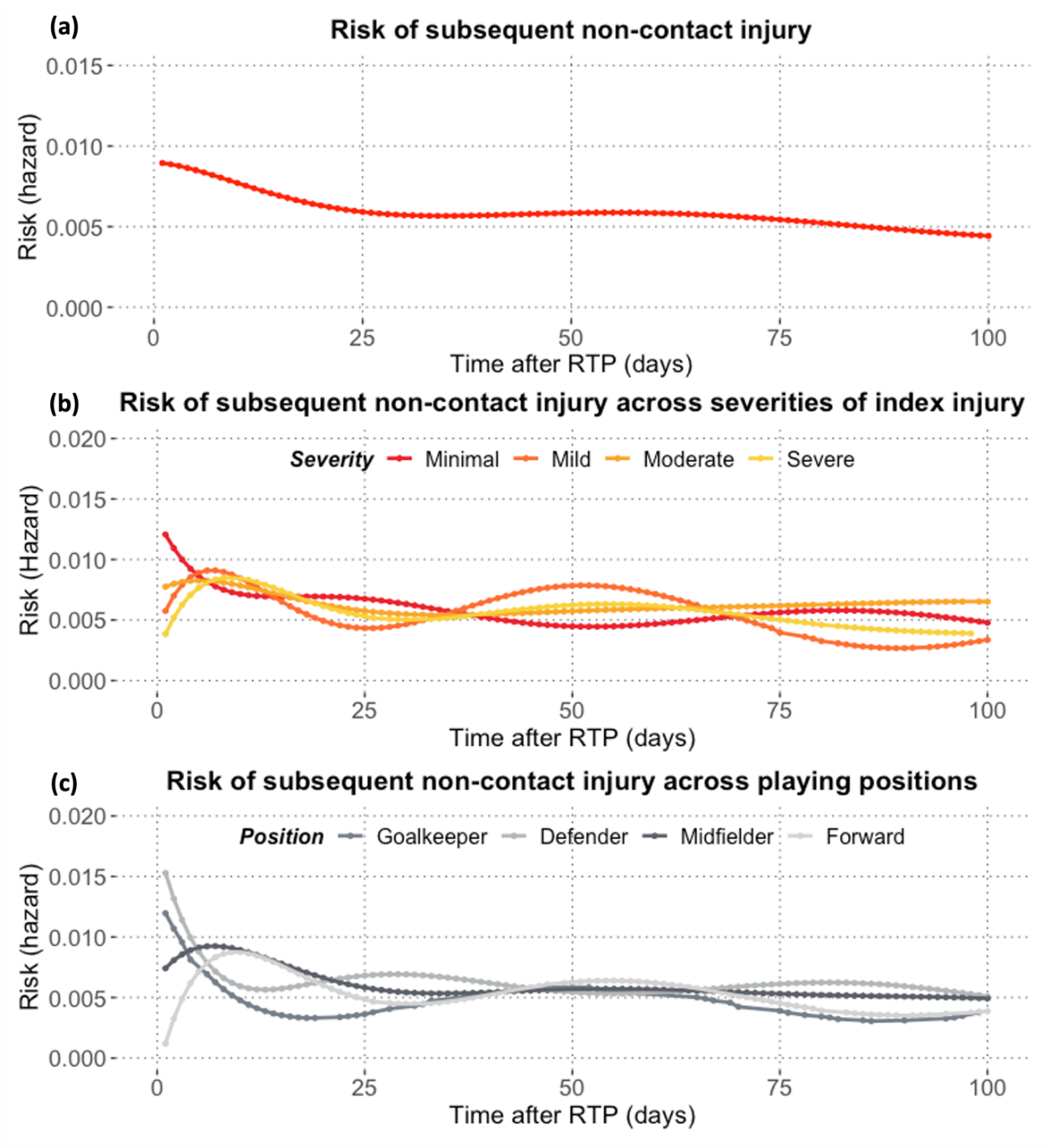
**

**Fig. S2-3** Based on up-sampled dataset, the time course of a) non-contact injury risk after RTP; non-contact subsequent injury risk across b) severities of index injury, and c) playing positions. RTP, return to play.

**Alternative data-processing 2: Including only the first RTP of each player within each season.**

**
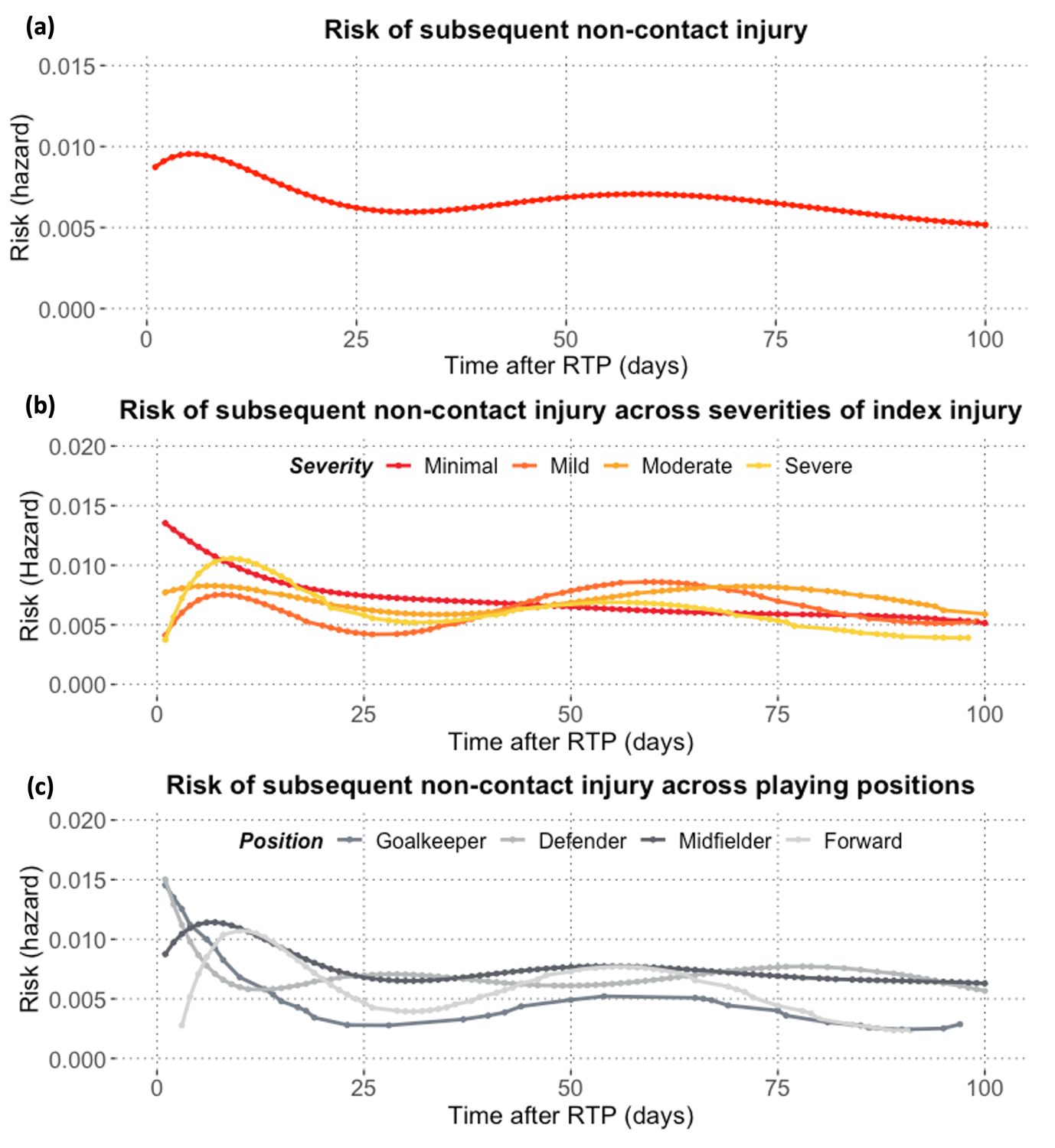
**

**Fig. S2-4** Based on the first RTP of each player within each season, the time course of a) non-contact injury risk after RTP; non-contact subsequent injury risk across b) severities of index injury, and c) playing positions. RTP, return to play.
