# Supplementary material for: The Time Course of Injury Risk After Return-to-Play in Professional Football (Soccer)
Source: Sports Med. 2024 Sep 14;55(1):193–201. doi: 10.1007/s40279-024-02103-3 (PMC11787231; doi:10.1007/s40279-024-02103-3)
Supplement: Supplementary file 3 — Data splitting for addressing overfitting concern (DOCX 2631 KB) [file 40279_2024_2103_MOESM3_ESM.docx]

**Probing potential overfitting: Comparing results for different subsets of the data**

The original dataset is split on the level of individual players (not episodes) into quarters in a round-robin manner. Distribution of playing positions is considered. Each quarter is held out in turn, leaving the remaining three quarters as subsets for analysis. Note that a split into several independent subsets, which would be preferable in principle, has not been possible due to the limited number of cases. This constraint has been particularly relevant for goalkeepers (10-11 players with 94-110 episodes) and for forwards (35-36 players with 94-110 episodes) in each of the four exclusive splits of the original data (twice that for splitting the dataset into two independent halves). A comprehensive overview is provided in table S3-1 below. Note that directly comparing these figures with the requirements of time-to-event analysis and the number of parameters to be fit (11 in the 10^th^ degree polynomial) is complicated by the hierarchy in the dataset (cp. ancillary analysis 2) and the sequential steps in the analytical pipeline (e.g., fitting cumulative hazards instead of raw data). However, the small size of the individual subsamples clearly favors spurious differences in results (variance). Therefore, out-of-population verification is warranted.

Results are illustrated in figure S3-1 below. Compared to the main analysis (left panel), similar patterns for global risk and risk across severities and playing positions are found in the four round-robin splits of the dataset (right panel below).

**Fig. S3-1** Time course of injury risk after RTP from a) main analysis, and b, c, d, e) four re-sampled datasets serving as a comparator. RTP, return to play.

| **Table S3-1** Description of subsets | | | | | |
| --- | --- | --- | --- | --- | --- |
| **Subsample** | **Positions** | **Players** | **Episodes** | **Severity**  **of index injury** | **Episodes** |
| 1 | Goalkeeper | 11 | 104 | Minimal | 979 |
|  | Defender | 59 | 796 | Mild | 482 |
|  | Midfielder | 68 | 1054 | Moderate | 596 |
|  | Forward | 35 | 452 | Severe | 349 |
| 2 | Goalkeeper | 10 | 109 | Minimal | 991 |
|  | Defender | 59 | 815 | Mild | 490 |
|  | Midfielder | 68 | 1084 | Moderate | 615 |
|  | Forward | 36 | 435 | Severe | 347 |
| 3 | Goalkeeper | 10 | 94 | Minimal | 999 |
|  | Defender | 59 | 808 | Mild | 506 |
|  | Midfielder | 68 | 1092 | Moderate | 571 |
|  | Forward | 35 | 436 | Severe | 354 |
| 4 | Goalkeeper | 10 | 110 | Minimal | 995 |
|  | Defender | 59 | 812 | Mild | 504 |
|  | Midfielder | 68 | 1130 | Moderate | 621 |
|  | Forward | 35 | 428 | Severe | 360 |
| The original dataset was split randomly on level of players stratified for playing position. | | | | | |
